# A dataset for dental anxiety and psychological distress in 1550 patients visiting dental clinics

**DOI:** 10.1186/s13104-021-05897-x

**Published:** 2022-01-10

**Authors:** Alexander Zinke, Christin Bohl, Hendrik Berth

**Affiliations:** Carl Gustav Carus Faculty of Medicine, Division of Psychological and Social Medicine and Developmental Neurosciences, Research Group Applied Medical Psychology and Medical Sociology, Technische Universität Dresden, Fetscherstr. 74, 01307 Dresden, Germany

**Keywords:** Dental anxiety, Stress, Psychological, Depression, Somatization, Anxiety, Adult, Dental clinic, Cross-sectional studies, Surveys and questionnaires, Objective

## Abstract

**Objectives:**

Information was collected to identify anxiety in dental patients visiting a dental clinic using the Dental Anxiety Scale, their level of psychological distress using the Brief Symptom Inventory-18 and identifying a correlation between these groups as well as the gender and age.

**Data description:**

This data contains a set of 1550 patients’ answers to questionnaires taken before dental treatment in a dental clinic. It is divided into male and female patients as well as according to their age. The level of Dental Anxiety can be interpreted by answers chosen in the Dental Anxiety Scale (DAS) and the level of psychological distress by answers chosen in the Brief Symptom Inventory-18 (BSI-18). This dataset should help to encourage more research in the field of dental anxiety and we hope to see more comparisons with our data in the future or in different regions of the world.

## Background

Dental anxiety is a very common anxiety disorder in the general population. It is alarming that even today 80% of all adults in industrial countries feel discomfort before dental treatment, 20% feel scared of it, whereas 5% evade it fully [1].

Dental treatment applies to all age groups, whereas anxiety and depression are more frequent in the younger patients [2] [3]. The focus of collecting this information was to provide a current database of questionnaires describing the emotional status of several patients linked to the following dental treatment. The Brief Symptom Inventory-18 (BSI-18) is a questionnaire first introduced by Derogatis [4] as a further shortened BSI, which contained 53 items out of the first Symptom-Checklist 90-R. It was originally developed as an instrument to define the state of psychological distress with only 18 items [5]. The Dental Anxiety Scale (DAS) was introduced by Corah in 1969 and today it is still one of the main instruments to assess dental fear in patients [6, 7]. It contains four questions referring to situations before and during dental treatment.

We hope that our dataset will be useful to the community of researchers outside Germany in order to analyze how and why these results are occurring in their countries.

## Data description

This data (Data file 1 in Table 1) [8] provides a set of 1550 patients that completed the Dental Anxiety Questionnaire (DAS) [6, 7], as well as the Brief Symptom Inventory-18 (BSI) [4, 5] before dental treatment. It was collected throughout the years 2012 to 2015 by the research group for Medical Psychology and Medical Sociology in Dresden (Germany). Data was collected in more than ten different dental clinics/surgeries in Germany. Only patients above the age of 18 years were included. Other inclusion criteria were sufficient knowledge of the German language, physical and mental ability to complete the questionnaires, mental orientation in space and time as well as no display of psychiatric symptoms. All patients gave written informed consent and were therefore included in the study. The questionnaires were administered while the patients were waiting for the treatment by the dentist. The data of a patient contains sex, age, DAS scores on questions 1 to 4 and the BSI score on questions 1 to 18.

**Table 1:** Overview of data files/data sets

Label	Name of data file/data set	File types (file extension)	Data repository and identifier (DOI or accession number)
Data file 1	DentalAnxiety.xlsx	MS Excel File (.xlsx)	OpARA—Open Access Repository and Archive Technische Universität Dresden, Technische Universität Bergakademie Freiberg https://dx.doi.org/10.25532/OPARA-131 [8]
Data file 2	Data description.pdf	Portable Document File (.pdf)	OpARA—Open Access Repository and Archive Technische Universität Dresden, Technische Universität Bergakademie Freiberg https://dx.doi.org/10.25532/OPARA-131 [8]

Out of all patients 56% are female. The mean age of the patients was 46 years.

The DAS questions are scored from 1 to 5, where “1” means no discomfort of the situation coming at all and “5” means total fear. Questions 1 and 2 assess situations before treatment while the patient is in the waiting room. Questions 3 and 4 describe situations during treatment.

The Brief Symptom Inventory-18 consists of 18 items. They can be divided into categories “Somatization” containing items 1,4,7,10,13,16, “Depression” containing 2,5,8,11,14,17 and “Anxiety” containing items 3,6,9,12,15,18. They describe a certain feeling and whether or not the patient can relate to this question during the last 6 days. It can be answered with 1 (“not at all”) to 4 (“extremely”).

Data file 2 [8, see Table 1] is a help file to describe each field belonging to data file 1. The description is additionally included as a separate sheet in the data file 1.

## Limitations

All questionnaires were completed by the patients themselves. It is therefore possible that some patients did not answer the questions truthfully and might have reduced the severity of their answers to avoid being singled out as a patient with dental anxiety. One should consider that the dataset is likely to underrepresent dental anxiety in the population as it would be less likely to include phobic and severally phobic patients who avoid dental treatment. The treatment the patients were expecting after their survey was not assessed with any questionnaire. Patients with acute pain might already be psychologically weakened, expecting more pain and, therefore, being afraid of the treatment more than someone waiting for a routine dental check-up.

## Data Availability

The data described in this Data note can be freely and openly accessed on OpARA—Open Access Repository and Archive Technische Universität Dresden, Technische Universität Bergakademie Freiberg. Please see Table 1 and references [8] for details and links to the data.

## Abbreviations

BSI-18: Brief Symptom Inventory
DAS: Dental Anxiety Scale

## References

[1] de Jongh A, ter Horst G (1993). What do anxious patients think? An exploratory investigation of anxious dental patients’ thoughts. Community Dent Oral Epidemiol.

[2] Zinke A, Hannig C, Berth H (2019). Psychological distress and anxiety compared amongst dental patients- results of a cross-sectional study in 1549 adults. BMC Oral Health.

[3] Zinke A, Hannig C, Berth H (2018). Comparing oral health in patients with different levels of dental anxiety. Head Face Med.

[4] Derogatis NL (2001). BSI 18, Brief Symptom Inventory 18: Administration, scoring and procedures manual.

[5] Franke GH, Jaeger S, Glaesmer H, Barkmann C, Petrowski K, Brähler E (2017). Psychometric analysis of the brief symptom inventory 18 (BSI-18) in a representative German sample. BMC Med Res Methodol.

[6] Corah NL (1969). Development of a dental anxiety scale. J Dent Res.

[7] Tönnies S, Mehrstedt M, Eisentraut I (2002). Die Dental Anxiety Scale (DAS) und das Dental Fear Survey (DFS) – Zwei Messinstrumente zur Erfassung von Zahnbehandlungsängsten. Z Med Psychol.

[8] Berth H. A dataset of 1550 patients to the questionnaires Dental Anxiety Scale (DAS) and Brief Symptom Inventory (BSI-18). OpARA - Open Access Repository and Archive Technische Universität Dresden, Technische Universität Bergakademie Freiberg. 2021. 10.25532/OPARA-131.

